# Effect of orthodontic forces on cytokine and receptor levels in gingival crevicular fluid: a systematic review

**DOI:** 10.1186/s40510-014-0065-6

**Published:** 2014-12-09

**Authors:** Priyanka Kapoor, Om Prakash Kharbanda, Nitika Monga, Ragini Miglani, Sunil Kapila

**Affiliations:** Department of Orthodontics, Faculty of Dentistry, Jamia Millia Islamia, New Delhi, 110025 India; Division of Orthodontics and Dentofacial Deformities, Centre for Dental Education and Research, All India Institute of Medical Sciences, New Delhi, 110029 India; Department of Orthodontics and Pediatric Dentistry, Graduate Orthodontics Program Director, The University of Michigan School of Dentistry, 1011 North University Avenue, Ann Arbor, MI 48109-1078 USA

**Keywords:** GCF, Cytokines, Chemokines, Receptors, IL-1β, RANK/RANKL, OPG

## Abstract

This systematic review aimed to generate evidence on role of potent markers of inflammation [cytokines, chemokines, their associated receptors and antagonists] following the application of orthodontic forces. Subsequent to registration with PROSPERO, literature search followed a predetermined search strategy to key databases along with hand search (HS). Seventy-seven articles from PubMed (P), 637 from Scopus (S), 51 from Embase (E), and 3 from hand search (HS) were identified. A total of 39 articles were shortlisted that met strict inclusion and exclusion criteria and quality assessment. Each study was evaluated for participant characteristics, study design, oral hygiene regimen, and gingival crevicular fluid (GCF) handling. Among these studies, biomarkers in the order of frequency were interleukin (IL)-1β (N = 21), tumor necrosis factor (TNF)-α (N = 10), IL-8,IL-6(N=8), receptor activator of nuclear factor kappa-B ligand (RANKL) (N = 7), monocyte chemoattractant protein (MCP)-1 (N = 3), IL-2 (N=4), IL-4, IL-10, RANTES (N = 2), IL-1, IL-5, IL-1α, IP-10, osteopontin (OPN) (N = 1) and receptors and their antagonists in the order of osteoprotegerin (OPG) (N = 8), IL-1RA (N = 5), and RANK (N = 1). Results revealed an immediate release of inflammatory bone-resorptive mediators, IL-1β and TNF-α, where IL-1β increased as early as 1 min to 1 h reaching peak at 24 h while TNF-α increased at 1 h or 1 day. This was accompanied by a fall in bone-protective mediator (OPG) levels at 1 h and 24 h after orthodontic force application. Continuous forces were accompanied by a decrease in mediator levels after attaining peak levels (most commonly at 24 h) while repeated activations in interrupted force upregulated their secretion. Significant correlations of IL-1β levels with pain intensity, rate of orthodontic tooth movement (OTM) and of activity index (AI) (IL-1β/IL-1RA) with velocity of tooth movement and growth status of individuals have also been deduced. A greater AI and RANKL/OPG ratio was seen in juveniles as compared to adults or non-growers that were associated with faster rate of OTM in juveniles. None of the studies addressed the effect of estrous cycle in female subjects. Lack of homogeneity in several parameters calls for a better controlled research on the biology of OTM.

**Electronic supplementary material:**

The online version of this article (doi:10.1186/s40510-014-0065-6) contains supplementary material, which is available to authorized users.

## Review

### Background

Orthodontic tooth movement (OTM) is considered an epiphenomenon of the gene expression of the periodontal ligament (PDL) and neighboring cells resulting from a series of orchestrated cellular and molecular events in alveolar bone and periodontal tissue initiated by the application of orthodontic force [1]. A chemical cascade that mediates the transmission of signals from extracellular matrix leading to genetic modulation is interceded by the release of mediators in paracrine environment. These signals are responsible for a change in the cytoskeletal structure, leading to alteration of nuclear protein matrix and eventually gene activation or suppression [2,3]. These events initiate the process of bone remodeling, leading to effective tooth movement. The biochemical mediators released sequentially at multiple stages during orchestration of tooth movement can be detected in gingival crevicular fluid (GCF). GCF is a unique biological exudate that has been found as a convenient medium to study these mediators with reasonable sensitivity. GCF can be collected noninvasively [4] with specifically designed filter paper or a micropipette (1 to 10 μl) or through magnetic beads placed in gingival crevice. GCF once collected may be cryopreserved or directly sent for chemical analysis. GCF can also be collected repeatedly at various stages of orthodontic treatment and therefore provides useful insight to biological events over the entire duration of observation.

Clinically demonstrable success of OTM is associated with expression of numerous regulatory molecules, of which cytokines have been most widely documented. Cytokines are low-molecular weight proteins (mw < 25 kDa) released in autocrine or paracrine environment in response to local signals like application of stress [5] and are involved in normal physiological bone turnover and remodeling [6-8]. Cytokine biology as a retort to forces applied for OTM is difficult to comprehend due to sheer number and complexity of these factors exhibiting redundancy as well as pleiotropy [9]. Although cytokines have been extensively evaluated in GCF as quantitative biochemical indicators of inflammatory periodontal status [10], there has been an increasing interest on understanding their contributions as mediators of OTM owing to their role in bone and tissue remodeling. Among these cytokines, interleukins (ILs) (IL-1α, IL-1β, IL-1RA, IL-8, IL-2, IL-6, and IL-15), tumor necrosis factors (TNFs), interferons (IFNs), growth factors (GFs), and colony-stimulating factors (CSFs) have been extensively studied in relation to OTM.

The secretion of these mediators in the local environment by cells activated on application of orthodontic force varies according to the force levels and functional state of available target cells. The rate of OTM depends on recruitment of mature osteoclasts and precursors, osteoclast differentiation and number of functional osteoclasts at the bone-PDL interface, where bone resorption is considered a rate-limiting step [11]. The earliest identified marker of bone resorption is IL-1β, closely followed by prostaglandin E2 (PGE2), nitric oxide, IL-6, and other inflammatory cytokines [12]. TNF is also believed to have synergistic effects with IL-1 [13]. Osteoclast differentiation and activation is mediated by the binding of receptor activator of nuclear factor kappa-B to its ligand expressed by osteoblasts and PDL cells (RANK and RANKL, respectively) [11,14-16]. This interaction and osteoclast activity is prevented by a decoy receptor for RANKL called osteoprotegerin (OPG) [17]. Thus, the relative balance between RANK-RANKL and OPG may be critical to the magnitude and rate of OTM.

The first experimental evidence supporting role of cytokines in OTM was documented in periodontal tissues of cat canine teeth where IL-1α and IL-1β were identified after the application of a tipping force [18]. One of the earliest noninvasive studies on IL-1β in GCF was done by Grieve et al. [19] where significant elevations from baseline in IL-1β and PGE levels supported initial release of proinflammatory mediators on application of orthodontic forces, followed by a decrease in 7 days (d). Since then, numerous studies in humans have focused on alterations in IL-1β levels in GCF in an attempt to understand the underlying inflammatory process during OTM. The studies have now diversified to include other interleukins like IL-1α, IL-2, IL-6, IL-8, and receptor antagonist IL-1RA. More recently, the presence of other mediators including TNF-α, TGF-β, leptin, RANK/RANKL, and OPG have also been explored in OTM.

This systematic review aims to determine effect of orthodontic forces on levels of markers of inflammation namely cytokines, chemokines, receptors and their antagonists, which have been widely documented in GCF. The present study looks into literature to generate evidence on the role of these mediators in relation to the force levels, applied mechanics, age, sex and pain intensity during orthodontic treatment. This systematic review provides insights into possible biomarkers for tooth movement and their potential contributions to modulating orthodontic bone turnover that could prove useful in designing future approaches to modulating OTM.

## Material and methods

**Figure 1:**
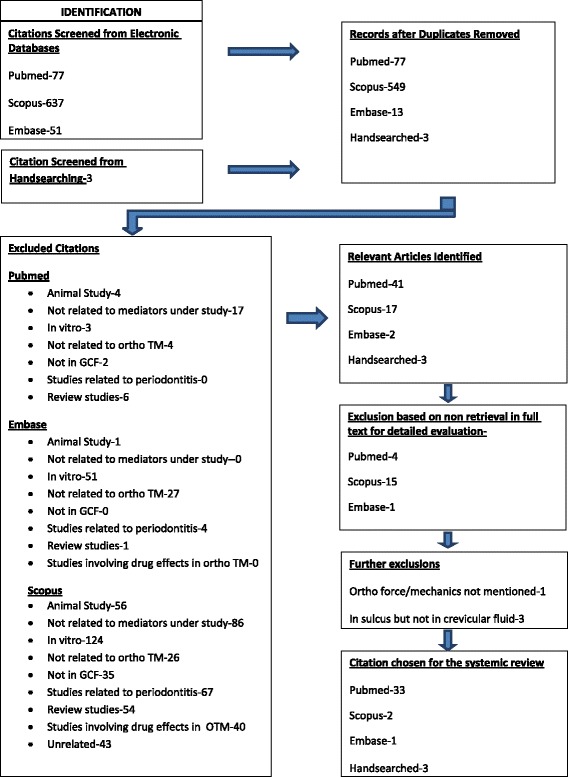
**Flowchart depicting the retrieval of studies for review process.**

This review was registered in PROSPERO on 3 June, 2014 (registration number CRD42014009302). The systematic review was conducted strictly adhering to guidelines suggested by PROSPERO. A search strategy was finalized utilizing MESH terms, Boolean terminology, and free text terms (Additional file 1: Annexure 1). This search strategy was applied to key databases PubMed, Scopus, and Embase in April 2014 by two reviewers PK and NM which followed a cross check by third reviewer OPK. Apart from that, hand search of journals was performed for article retrieval. Segregation of articles to be considered for review was based on stringent inclusion and exclusion criteria. Seventy-seven articles in PubMed, 637 in Scopus, 51 in Embase, and 3 from hand searching were retrieved (Figure 1). Duplicates were removed before final screening of articles for inclusion in the review. The titles and abstracts of these manuscripts were studied, considering exclusion and inclusion criteria specific to each database (Figure 1). The relevant articles were obtained. These were PubMed - 41, Scopus - 17, Embase - 2 and hand searched - 3. Further, a few articles had to be excluded for non-availability of full text. These were PubMed (N = 4), Scopus (N = 15), and Embase (N = 1). Further, four articles had to be excluded since full text revealed no mention of orthodontic force (N = 1) or study was not performed in crevicular fluid (N = 3). A consensus has arrived among reviewers for final inclusion of 39 articles comprising of 33 articles from PubMed, 2 from Scopus, 1 from Embase, and 3 from hand search (Figure 1).

**Table 1:** **Participant characteristics**

**Ref no.**	**Sx**	**M/F**	**Age**	**Mediators**	**Index T**	**Cont T**	**Site sp**	**Rnd**	**Mal**	**N drug H**	**N bone Ls**	**N Ging inflm**	**PD (<2 mm)**
22	16	2 M, 14 F	18 to 24 y	IL-1β	Mx and Md C	Md R or L C	D	Y	Class I biMx	Y	NM	Y	NM
23	22	11 M, 11 F	14.4 + _1.1 y	Leptin	Mx C	Contr C	D	N	Mx 1st PM Extr cs	Y	Y	Y	Y
24	33	21 M, 12 F	10.8 to 30.9 y	IL-1β, IL-1RA	Mx C	IP Md C/Aj T	D	y	Mx 1st PM Extr cs	NM	NM	NM	NM
25	12	7 F, 5 M	13 to 17 y	OPG	Mx C	Contr C	D	N	Mx 1st PM Extr cs	Y	Y	Y	Y
26	9	5 M, 4 M	10 to 18 y	IL-1β, βG	1st Mo, 1st PM, CI	NM	MP and MB	N	RME cs	NM	NM	NM	NM
27	12 SD (6) LD (6)	SD: 3 M, 3 F LD-NM	SD: 11 to 18 y LD: 19 to 27 y	IL-1β, −6, IL-8, TNF-α	Mx 1st PM	Ant T	DB	N	Mx 2nd PM Extr cs	Y	Y	Y	Y
28	10	4 M, 6 F	NM	TNF-α	C	NM	D	N	Mx 1st PM Extr cs	Y	Y	Y	Y
29	18	9 M, 9 F	16 to 19 y	IL-1β, −TNF-α	Mx C	NM	M and D	N	1st PM Extr cs	Y	Y	Y	Y
30	30:15 J/15 A	J: 7 M, 9 F A: 6 M, 9 F	J: 15.1 ± 2.8 y	RANKL, OPG	1 Mx C	Contr and ant C	D	N	Mx 1st PM Extr cs	Y	Y	Y	Y
			A: 31 ± 3.6 y										
31	15	6 M, 9 F	15 to 19 y	IL-2, IL-6, IL-8	Mx C	NM	M and D	N	1st PM Extr cs	Y	Y	Y	Y
32	10	4 M, 6 F	M −14.5 y	RANKL, OPG	Mx C	Contr and opposing C	D	N	Mx 1st PM Extr cs	Y	Y	Y	Y
			F 15.4 y										
33	18	10 M, 8 F	8.9 to 13.8 y	IL-1β, SP, PGE2	1st Mx/Md M	Ant L or R 1st M	DB and DP	N	Crowding in 1 or both jaws	Y	Y	Y	Y
34	9	3 M, 6 F	M: 21.3 ± 2.8 y	IL-1β, SP	Mx C	Contr C	D	N	Mx 1st PM Extr cs	Y	Y	Y	Y
			F: 23.1 ± 2.4 y										
35	10	3 M, 7 F	10 y 5 m to 30 y 11 m	IL-1β, IL-1RA	R and L Mx C	Md. R C	M and D	Y	Mx 1st PM Extr cs	NM	NM	NM	NM
36	10	NM	15 to 17 y	IL-8	Mx and Md C	NM	M and D		1st PM Extr cs	Y	Y	Y	Y
37	10	2 M, 8 F	18.4 to 22.5 y	IL-1β, PGE2	Mx C (E1), Contr Mx C (E2)	Ant Md C	D	N	All 1st PM Extr cs	Y	Y	Y	Y
38	84	43 J (M)	J: 11 ± 0.7 y	PGE-2, IL-6, GMCSF	Mx LI	Contr LI	M and DB	N	Labial tipping reqd	Y	Y	Y	Y
		41 A (M)	A: −24 ± 1.6 y										
39	9	5 M, 4 F	10 to 18 y	IL-1β βG	1st Mo, 1st PM, CI	NM	MP and MB	N	RME cs	Y	Y	Y	Y
40	7	2 M, 5 F	12 y 3 m to 16 y 3 m	IL-1β, IL-1RA, AI	Mx C	Md C	M and D	NM	All 1st PM Extr	Y	NM	Y	NM
41	12	3 M, 9 F	14.4 ± 0.9 y	TGF-β	C	Ant C/Contr C	D	NM	All 1st PM Extr	Y	Y	Y	Y
42	12	3 M, 9 F	14.4 ± 0.9 y	IL-1β, IL-6, TNF-α, EGF, β2-μG	C	Ant C/Contr C	D	NM	All 1st PM Extr	Y	Y	Y	Y
19	10	5 M, 5 F	M: 24.6 ± 1.5 y F: 27.8 ± 3.9 y	IL-1β, PGE	Mx LI, Mx 1st PM	Contr T	MB	N	Buccal/labial OTM	Y	Y	Y	Y
43	50	23 M, 27 F	13 to 20 y	IL-1β, TNF-α, NO	Mx I, Mx C	NM	M and D	NM	Non Extr	Y	NM	Y	Y
44	10	5 M, 5 F	12 to 16 y	RANKL, OPG	1st PM (quad 1)	1st PM (quad 2)	D	NM	2nd Mx PM Extr	Y	NM	Y	NM
45	22	7 M, 15 F	19 to 29 y	HSP70, RANKL	Mx C	Contr C		NM	Mx 1st PM Extr	Y	NM	Y	NM
46	12	6 M, 6 F	11 to 15 y	MMP-8, IL-1β	1st M, CI, C	NM	M	NM	NM	Y	Y	Y	Y
47	21	NM	12 to 20 y	GM-CSF, IF-ϒ, IL-1β, IL-2, IL-4, IL-5, IL-6, IL-8, IL-10 and TNFα, MMP-9, TIMP-1 and 2, RANKL, OPG	Mx C	2nd M	MB and DP	NM	Mx 1st PM Extr	Y	Y	Y	Y
48	10	5 M, 5 F	22 to 29 y	RANK, OPG, OPN, TGF-β1	1st M	Contr M	Exp T: MB and DB Cont T: MB and ML	Y	NM	Y	NM	Y	Y
49	14	3 M, 11 F	12 to 28 y	MMP-3, MMP-9, MMP-13, MIP-1β, MCP-1, RANTES	Mx C	NM	M and D	NM	Mx 1st PM Extr	Y	NM	NM	NM
50	18	8 M, 10 F	8.9 to 13.8 y	IL-1β, SP, PGE2	1st M	Contr M	M and D	NM	Crowding in Mx and Md	Y	Y	Y	Y
51	10	NM	16.3 ± 2.5 y	IL-1β	Mx C	Md C	DB	NM	Class II, all 1st PM Extr	Y	Y	Y	Y
52	16	8 M, 8 F	16.6 ± 2.4 y	IL-2, IL-6, IL-8	Mx C	Mx 2nd PM	DB	NM	Mx 1st PM Extr	Y	Y	Y	Y
53	20	C: 3 M, 7 F EX:5M,5 F	18 to 45 y	CCL-2 (MCP1), CCL-3, CCL-5 (RANTES), IL-8 (CXCL8), IL-1α, IL-1β, IL-6, TNF-α	Mx C	Contr C	DB	Y	Class II Div 1 Mal With 1st PM Extr	Y	Y	Y	Y
54	16	8 M, 8 F	16.6 ± 2.4 y	TNF-α	Mx C	Mx 2nd PM	DB	NM	Mx 1st PM Extr	Y	Y	Y	Y
55	17	9 M, 8 F	16 to 20 y	TNF-α, IL-1β, IL-8	Mx C	NM	M and D	NM	All 1st PM Extr	Y	Y	Y	Y
56	10	5 M, 5 F	15 y ± 3 y 8 m	IL-1β, IL-1RA	Mx C	Md C/Aj t	D	NM	All 1st PM Extr	NM	NM	NM	NM
57	20	10 ado (3 M, 7 F), 10 A (4 M, 6 F)	Ado: 14.4 ± 1.43 y A: −28.5 ± 7.83 y	RANKL, OPG, IL-1, IL-1RA, MMP-9	Mx I	Md I	Lab	NM	Non Extr, 3 to 6 mm I crowding	N	N	Y	N
58	24 HG-14 NHG-10	10 M, 14 F HG: 6 M, 8 F	14.66 ± 1.1 y	IL-2, IL-4, IL-6, IL-8, IL-10, GM-CSF, IFN-γ, TNFα, MCP-1, IP-10	Mx 1st M and Mx 1st PM	NM	MB and DB	N	Non extr cs	Y	NM	Y	Y
59	9	4 M, 5 F	17.5 to 18.9 y	OPG, RANKL	Mx C	NM	M and D	NM	All 4 Extr	Y	Y	Y	Y

*Ref No.* reference number, *S* sample, *M/F* male/female, *med* mediator, *T* tooth, *sp* specification, *Rnd* randomization, *Mal* malocclusion, *HS* hand searched, *P* PubMed, *S* Scopus, *E* Embase, *N* no, *Y* yes, *Mx* maxilla, *Md* mandible, *H* history, *Ls* loss, *Ging* gingival, *Inflm* inflammation, *PD* probing depth, *NM* not mentioned, *m* month, *d* day, *wk* week, *h* hour, *R* right, *L* left, *C* canine, *PM* premolar, *Mo* molar, *CI* central incisor, *Ant* antagonistic, *Contr* contralateral, *IP* interproximal, *oppos* opposing, *Exp* experimental tooth, *Cont* control tooth, *Aj* adjacent, *E1* experimental site 1, *E2* experimental site 2, *Extr* extraction, *M* mesial, *D* distal, *Retr* retraction, *y* year, *cs* cases, *IL* interleukin, *NO* nitric oxide, *RANKL* receptor activator of NFкB ligand, *β2-μG* β2 microglobulin, *TNF* tumor necrosis factor, *TGF* transforming growth factor, *EGF* epidermal growth factor, *SP* substance P, *PGE* prostaglandin, *HSP* heat shock protein, *IFN* Interferon, *MMP* matrix metalloproteinase, TIMP tissue inhibitor of metalloproteinases, *MCP* monocyte chemoattractant protein, *MIP* macrophage inflammatory process, *RANTES* regulated on activation normal T cells expressed and secreted, *GM-CSF* granulocyte-macrophage colony-stimulating factor, *reqd* required, *quad* quadrant, *OTM* orthodontic tooth movement, *Lab* labial surface, *ado* adolescent, *J* juveniles, *A* Adults.

**Table 2:** **Study characteristics**

**Ref no.**	**F**	**T/O F**	**Mech**	**Mech/O appli**	**React**	**Tot Du**	**No. of obs**	**Time obs**	**Bas**	**Bas (same as cont)**
22.	50 and 150 g	Cont F	Retr- se	Ni-Ti spg	N	2 m	6	0, 1, 24 h, 1 wk, 1 m, 2 m	0	N
23.	250 g	Intrrup F	Retr- se	E-chain	NM	168 h	4	0, 1 h, 24 h, 168 h	0	N
24	4, 13, 26, 52, or 78 kPa	Cont F	Retr- se	Vert loop act. with Ni-Ti spg	N	84 d	9 to 10	0, 1, 3, ±7, 14, 28, 42, 56, 70, 84 d	0	N
25	150 g	Cont F	Retr- se	Ni-Ti spg	N	3 m	6	before act 0, after act 1 h, 24 h, 168 h, 1 m, 3 m	0	N
26	NM	Interm F	Mx expans	Hyrax screw	Y	81 d	10	0, 14, 25, 32, 33, 39, 46, 53, 60, 81 d	14 d	Y
27	SD-NM LD-50 cN	SD - intrrup F LD - cont F	SD - space gaining LD - Retr- se	SD-E - separt LD - Ni-Ti spg	N	SD - 24 h LD - 4 m	SD - 2 LD - 5	SD - 0 h, 24 h LD - 0, T1, 1 m, 2 m, 3 m	0	N
28	HG - RDG-NM	HG - cont F RDG - heavy Intrrup F	Retr- se	HG - hybrid retract RDG - C distalizer	HG-N RDG-Y	1 wk	4	Before act (0), after act 1 h, 24 h, 1 wk	0	Y
29	Level-NS	Cont F	Level Retr- se	0.014” NiTi wire Sentalloy c.c. spg	N	6 m 21 d	7	Level - 0, 7 d, 21 d, 3 m After 6 m Retr - 6 m (0), 7 d, 21 d	Level - 0 Retr - 6 m-0	Y
	Retr - 150 g	Cont F								
30	250 g	Intrrup F	Retr- se	E-chain	NM	168 h	4	0, 1, 24, 168 h	0	N
31	Level-NS	Cont F	Level Retr- se	0.014” NiTi wire Sentalloy c.c. spg	NM	6 m 21 d	6	Level - 0, 7 d, 21 d After 6 m Retr - 6 m (0), 7 d, 21 d	Level - 0 Retr - 6 m-0	Y
	Retr - 150 g	Cont F								
32	250 g	Intrrup F	Retr- se	E-chain	N	7 d	4	0, 1, 24, 168 h	0	N
33	NM	Intrrup F	Space gaining	E - separt	N	14 d	5	−7 d, 0 d, 1 h, 1 d, 7 d	0	N
34	250 g	Intrrup F	Retr- se	E-chain	N	168 h	8	0, 1, 4, 8, 24, 72, 120, 168 h	0	N
35	60, 18, 120, 240 g	Cont F	Retr- se	Vert loop act. with Ni-Ti spg	N	112 d	11	−28, −14, 0, 1, 3, 14, 28, 42, 56, 70, 84 d	0	N
36	Mx C - 115 g	Cont F	Retr- se	Ricketts seg arch	N	30 d	6	0, 1 h, 24 h, 6 d, 10 d, 30 d	0	Y
	Md C - 90 g									
37	E1: 100 g	E1: Cont	Retr	E1: NiTi spg	E1: N	3 wk	10	0, 1 h, 24 h, 1 wk, repeat twice	0	N
	E2: NM	E2: Intrrup		E2: screw attached retractor	E2: 2					
38	70 cN	Cont	Tipping	bu/la offset	N	24 h	2	0, 24 h	0	N
39	NM	Interm F	Mx expans	Hyrax screw	Y	81 d	11	0, 14, 18, 25, 32, 33, 39, 46, 53, 60, 81 d	0	Y
40	13 to 4 kPa	Cont	Retr	V loop act by spg	N	84 d	9	0, 1, 3 d, 14 d intervals until 84 d	0	N
41	2 to 2.5 N	Intrrup	Retr	E-chain	N	7 d	4	0, 1, 24, 168 h	0	N
42	250 g	Intrrup	Retr	E-chain	N	7 d	4	0, 1, 24, 168 h	0	N
19	100 g	Cont	Labial tipping	La offset in NiTi wire	N	7 d	5	0, 1, 24, 48, 168 h	0	N
43	NM	NM	NM	Alignment	NM	6 m	3	0, 1 m, 6 m	0	N
44	150 g	Cont	Retr	NiTi spg	N	45 d	6	0, 2, 4, 7, 30, 45 d	0	N
45	130 g	Intrrup	Retr	E-chain	N	24 h	2	0, 24 h	0	N
46	NM	NM	NM	Bracket placement	N	3 m	4	0, 24 h, 1 wk, 3 m	0	Y
47	100 g	Cont	Retr	NiTi c.c. spg	N	42 d	4	−10 wk, 0, 4 h, 7 d, 42 d	0	N
48	NM	Intrrup	Space gain	Elastic separt	N	7 d	3	0, 24 h, 7 d	0	N
49	150 g	Cont	Retr	V - loop and NiTi c.c. spg	N	87 d	7	−7 d, 0, 1 h, 24 h, 14, 21, 80 d	0	Y
50	NM	Intrrup	Space gin	Elastic separt	N	14 d	6	−7 d, 0, 1 min, 1 h, 1 d, 7 d	0	N
51	120 g	Cont	Retr	NiTi c.c. spg	N	21 d	6	1 h, 24 h, 48 h, 168 h, 14 d, 21 d	1 h	N
52	150 g	Cont	Retr	Sentalloy c.c. spg	N	3 m	7	0, 1, 24, 48 h, 7 d, 21 d, 3 m	0	N
53	100 g	Cont F	Retr- se	Ni-Ti c.c. spg	N	28 d	4	0, 1 d, 7 d, 28 d	0	Y
54	150 g	Cont	Retr	Sentalloy c.c. spg	N	3 m	7	0, 1, 24, 48 h, 7 d, 21 d, 3 m	0	N
55	NM	Cont	Alignment	0.014 NiTi wire	N	7 d	8	0, 1, 2, 3, 4, 5, 6, 7 d	0	Y
56	13, 26, 52 kPa	Cont	Retr	V loop act by spg	N	84 d	9	0, 1, 3 d, 14 d intervals until 84 d	0	N
57	NM	Cont	Alignment	0.014 - NiTi, 0.016 × 0.022 NiTi, 0.019 × 0.025 NiTi	N	20 wk	6	0, 3 wk, 6 wk, 12 wk, 2 wk	0	N
58	NM	HG: band intrrup F Bond - Cont F NHG - Cont F	Level	0.014 - in NiTi wire 0.016 × 0.022-in s.s.	N	70 wk	HG: band-3 HG; bond-2 NHG-2	HG: band - 18, 0, 52 wk	HG: band - 18 wk	Y
								HG: bond - 0, 52 wk	Bond - 0 wk	
								NHG - 0, 52 wk	NHG - 0 wk	
59	200 g	Cont	Retr	Sentalloy c.c. spg	N	1 m	5	0,1 h, 24 h, 168 h, 1 m	0	Y

*Ref No.* reference number, *F* force, *T/O* type of, *Mech* mechanics, *Mech/O* mechanics of, *appli* appliance, *React* reactivation, *Tot* total, *Du* duration, *N* number, *obs* observation, *Bas* baseline, *min* minutes, *g* grams, *Intrrup* interrupted, *Cont* continuous, *interm* intermittent, *Retr* retraction, *se* segmented, *spg* spring, *E-chain* elastomeric chain, *NiTi* nitinol, cont control, *NM* not mentioned, *y* year, *d* day, *m* month, *h* hour, *Level* leveling, *separt* separator, *act* activated, *HG* headgear, *NHG* non-headgear, *bu* buccal, *la* labial, *HG* hybrid retract, *RDG* rapid canine distalizer.

**Table 3:** **Oral hygiene regimen and assessment of gingival health**

**Ref no.**	**Mu wsh**	**Freq/O Mu wsh/d**	**Oral prophy (pre t/t)**	**Oral prophy (every obser pt)**	**Asses for ging and perio inflam (pre t/t)**	**At every obser pt**
22	Chlorhex	Twice	NM	NM	Y	Y
23	N	NA	Y	Y	Y	At 0 and 168 h
24	NM	NM	Y	Y	Y	Y
25	NM	NA	Y	Y	N	N
26	Chlorhex	Twice	Y	NM	Y	Y
27	Chlorhex	Twice	Y	Y	Y	Y
28	NM	NA	NM	NM	Y	NM
29	NM	NA	NM	NM	NM	NM
30	NM	NA	Y	Y	Y	Y
31	NM	NA	NM	NM	NM	NM
32	NM	NA	Y	Y	Y	Y
33	Chlorhex	Twice	Y	Y	Y	Y
34	NM	NA	Y	Y	Y	Y
35	Chlorhex	Twice	Y	Y	Y	Y
36	NM	NM	Y	Y	Y	Y
37	NM	NM	NM	NM	Y	Y
38	NM	NM	Y	Y	NM	NM
39	Chlorhex	Twice	Y	NM	Y	Y
40	Chlorhex	Twice	Y	Y	Y	Y
41	NM	NM	NM	NM	Y	Y
42	NM	NM	NM	NM	Y	Y
19	Chlorhex	NM	NM	NM	Y	
43	NM	NM	Y	Y	Y	Y
44	Chlorhex	Twice	Y	Y	Y	Y
45	NM	NM	Y	Y	NM	NM
46	NM	NM	Y	Y (6 wk, 3 m)	Y	Y
47	NM	NM	NM	NM	Y	Y
48	NM	NM	Y	Y	Y	Y
49	Chlorhex	Twice	Y	Y	NM	NM
50	NM	NM	Y	N	Y	Y
51	NM	NM	NM	NM	NM	NM
52	NM	NM	Y	Y	Y	NM
53	NM	NM	Y	Y	Y	NM
54	NM	NM	Y	Y	Y	NM
55	NM	NM	NM	NM	NM	NM
56	Chlorhex	Twice	Y	Y	Y	Y
57	NM		Y	NM	NM	NM
58	NM	NA	Y	NM	Y	Y
59	NM		Y	Y	Y	Y

*Ref No.* reference number, *Mu* mouth, *wsh* wash, *Freq/O* frequency of, *d* day, *prophy* prophylaxis, *t/t* treatment, *obser* observation, *pt* point, *Asses* assessment, *ging* gingival, *perio* periodontal, *inflam* inflammation, *chlorhex* chlorhexidine, *Y* yes, *NM* not mentioned, *N* no, *h* hour.

**Table 4:** **GCF characteristics**

**Ref no.**	**Time**	**Temp**	**Humd**	**Site sp**	**Inser (in mm)**	**Rep meas**	**I/O meas**	**Meth/O coll**	**Du/O meas**	**Temp of sto**	**Meth/O meas**	**Anal meth**	**Prot conc**
22	NM	NM	NM	D	1 mm	2	90 s	PP	30 s	−80°C	PT8000	ELISA	pg/mg
23	Y	NM	NM	D	1 mm	4	60 s	PP	30 s	−80°C	PT8000	ELISA	pg/ml
24	NM	NM	NM	D	NM	2	NM	NM	NM	NM	NM	ELISA	NM
25	NM	NM	NM	D	1 mm	N	NA	PP	30 s	−80°C	PT8000	ELISA	pg/μl
26	NM	Y	Y	M	NM	N	NA	PP	30 s	NM	PT6000	ELISA	pg/30 s
27	NM	NM	NM	DB	1 mm	N	NA	PP	30 s	−20°C	PT8000	MB-IA	pg/μl
28	Y	NM	NM	D	1 mm	4	60 s	PP	30 s	−80°C	Elec scale	ELISA	pg/μl
29	Y	NM	NM	M and D	1 mm	N	NA	PP	30 s	−20°C	PT8000	IA	pg
30	NM	NM	NM	D	1 mm	2	60 s	PP	60 s	−30°C	PT8000	ELISA	pg/μl
31	Y	NM	NM	M and D	NM	N	NA	PP	30 s	−20°C	PT8000	IA	pg
32	NM	NM	NM	D	1 mm	1	60 s	PP	60 s	−30°C	PT8000	ELISA	pg/μl
33	NM	NM	NM	DB and DP	1 mm	NM	NA	DuFM	20 s	−70°C	NM	ELISA	pg/20-s samp
34	NM	NM	NM	D	1 mm	2	NM	PP	60 s	−30°C	PT8000	ELISA	pg/μg
35	NM	NM	NM	M and D	1 mm	1	60 s	PP	30 s	−70°C	NM	ELISA	mg/l - tot prot, IL-1-ng/g IL-1RA- μg/g
36	NM	NM	NM	M and D	1 mm	1	60 s	PP	30 s	−70°C	Elect scale	ELISA	pg/ml
37	Y	Y	Y	D	1 mm	4	1 min	PP	30 s	−70°C	NM	ELISA	pg/μg
38	NM	Y	NM	MB and DB	1 mm	N	N	PP	30 s	−80°C	PT6000	RIA	pg/μl
39	NM	Y	Y	M	NM	N	NA	PP	30 s	NM	PT6000	ELISA	pg/30-s GCF
40	NM	NM	NM	M and D	1 mm	1	1 min	PP	30 s	−70°C	NM	ELISA	Tot prot - mg/l, IL-1β (ng/g), IL-1RA (μg/g)
41	NM	NM	NM	D	1 mm	1	1 min	PP	30 s	−30°C	PT	ELISA, EP, WB	pg/μg
42	NM	NM	NM	D	1 mm	1	1 min	PP	30 s	−30°C	PT	ELISA	pg/μg
19	Y	Y	y	MB	1 mm	N	N	PP	30 s	−80°C	PT6000	RIA	pg
43	NM	NM	NM	M and D	NM	NM	NM	PP	30 s	NM	PT8000	ELISA	NM
44	NM	NM	NM	D	NM	N	N	PP	30 s	−80°C	PT8000	ELISA	pg/μl
45	NM	NM	NM	D	2 mm	1	2 min	PP	1 min	−20°C	NM	ELISA, WB, SDS-PAGE	NM
46	NM	NM	NM	M	NM	N	N	FP	3 min	−30°C	NM	ELISA	IL-1β; pg/ml, MMP-8: ng/ml
47	NM	NM	NM	MB and DP	NM	N	N	PP	30 s	NM	PT8000	LMAT	pg/ml
48	NM	NM	NM	ExpT: MB and DB Cont T: MB and ML	1 mm	N	N	PP	30 s	NM	PT8000	ELISA	Cyt conc - pg/μl Tot prot (pg)
49	NM	NM	NM	M and D	1 mm	N	N	PP	NM	−80°C	PT8000	MB-IA	pg/site
50	NM	NM	NM	MB and DB	1 mm	N	N	FM	20 s	−70°C	NM	ELISA	pg/20 s
51	Y	Y	Y	DB	NM	N	N	FP	3 min	−70°C	NM	ELISA	pg/μl
52	Y	NM	NM	DB	NM	1	1 min	PP	30 s	−20°C	PT8000	ELISA	pg/μl
53	Y	NM	NM	DB	1 mm	0	NA	FP	10 s	NM	PT8000	CPA	pg/μl
54	Y	NM	NM	DB	NM	1	1 min	PP	30 s	−20°C	PT8000	IA	pg/μl
55	Y	NM	NM	NM	1 mm	N	N	PP	30 s	−20°C	PT8000	IA	pg
56	NM	NM	NM	D	1 mm	1	1 min	PP	30 s	−70°C	NM	ELISA	Tot prot - mg/l, IL-1β (ng/g), IL-1RA (μg/g)
57	NM	NM	NM	DL	1 mm	N	N	PP	30 s	−80°C	NM	QAK	pg/ml
58	NM	NM	NM	MB and DB	NM	2	NA	PP	30 s	−70°C	PT6000	BHCA	pg/ml
59	NM	NM	NM	M and D	1 mm	1	1 min	PP	30 s	−70°C	Precisa 62 A	ELISA	pmol/l

*Ref No.* reference number, *Humd* humidity, *sp* specification, *Inser* insertion, *MB* mesio-buccal, *ML* mesio-lingual, *DP* disto-palatal sites, *DB* disto-buccal sites, *M* mesial, *D* distal, *NM* not mentioned, *N* no, Y yes, *PP* periopaper, *PT* periotron, *FP* filter paper strips, *FM* Durapore filter membrane, *WB* Western blot, *ELISA* enzyme linked immune sorbent assay, *SDS-PAGE* sodium-dodecyl sulfate polyacrylamide gel electrophoresis, *RIA* radio-IA, *I/O* interval of, *Meth/O* method of, *coll* collection, *meas* measurement, *Du/O* duration of, *Temp* temperature, *sto* storage, *Anal* analysis, *Prot* protein, *conc* concentration, *pg* picogram, *mg* microgram, *ml* milliliter, *μl* microliter, *GCF* gingival crevicular fluid, *tot* total, *g* gram, *ng* nanogram, *s* seconds, *min* minutes, °C degree Celsius, *elect* electronic, *IA* immunoassay, *EP* electrophoresis, *sP* spectrophotometry, *Ar* array, *A* assay, *MB* multiplex bead, *LMAT* Luminex multianalyte technology, *BHCA* Bio-Plex human cytokine assay, *CPA* custom protein array, *QAK* Quantibody Ar kit, *DuFM* Durapore filter membrane.

**Table 5:** **Result characteristics**

**Ref no.**	**Mediators**	**Stats analy appld**	**Confd**	**Drop outs**	**Up/down reg**	**Pk**	**Sec outcm**	**r**	**Stat sign readings**
22.	IL-1β	ANOVA and Friedman and paired *t*	Y	NM	Inc	24 h, 2 m	1. Mean tot prot conc - 12 mg/ml	C mov with less pain and inflam with 50 g than with 150 g of F	Inc at 24 h and 2 m in 150 g F compd to cont
							2. VAS score of 150 g > 50 g at 24 h		
23.	Leptin	Wilcoxon, Friedman	NM	NM	Dec	168 h	NM	NM	b/w bas and 168 h in exp T
24	IL-1β, IL-1RA	ANCOVA	Y	Y	Fluct	NM	For same stress and grw status, max diff in speed were 4.2:1 for 13 kPa in growers and 4.8:1 for 26 kPa in Nn-growers	Higher speeds of T move sign assoc with gen type 2 at IL-1β (+3,954), higher AI, and lower IL-1RA in GCF	NM
25	OPG	Shapiro Wilk’s Normality, Wilcoxon, Friedman, Z	NM	NM	Dec	NM	NM	NM	Dec at 1 h, 24 h, 168 h, 1 m, 3 m compd with bas
26	IL-1β, βG	1-tailed paired Student *t*	Y	NM	Inc	M-010 PM-07 CI-08	βG sign inc for M - 07 to 010 PM-07,08,010 CI - 06, 07, 010 and dec at O2 for M, PM, CI	Stronger F cause higher levels of both IL-1β and βG	IL-1β sign inc for M - O5 to O10 for PM-O6 to 010. For CI - 04, 06, 07, 09, 010 and dec at O2 for M, PM, CI
27	IL-1β, IL-6, IL-8, TNF-α	Mann-Whitney, Kruskal-Wallis	Y	NM	Inc	SD - 24 h LD - T1	NM	NM	In SD IL-1β, IL-8, TNF-α inc, In LD inc of IL-8 at T1
28	TNF-α	Intergrp - Mann-Whitney U Intragrp - Wilcoxon signed rank	NM	NM	Inc	24 h	PI, PD, BOP sign higher in HG GCF vol at 1 h and 24 h in the RDG > HG	NM	HG - a stat sign dec at 1 wk compd to 24 h. RDG - inc at 1 h stat sign compd to initial value. Inc in RDG at 1 h > HG
29	IL-1β,-TNF-α	1-way paired *t*, Mann-Whitney U	NM	NM	Inc	7 d, 21 d	GCF vol inc on 7 d and 21 d of level and retr	NM	TNF –α diff b/w 3 m (level) and 6 m (bf retr)
30	RANKL, OPG	3-way analysis of variance Tukey’s honest sign diff	Y	NM	RANKL- Inc OPG- Dec	24 h	avg amt of TM for J > A after 168 h mean vol of GCF in A sign lower than J	GCF vol correl with inflam state	RANKL at 24 h - sign inc levels both in J & A. RANKL and OPG in A < J OPG at 24 h sign dec levels both in J & A. RANKL/OPG for exp T sign > cont after 24 h RANKL/OPG in A < J
31	IL-2, 6, 8	1-way paired *t* Mann-Whitney U	NM	NM	IL-2-inc	IL-2 - 7 d, 21 d of level IL-8 - 7 d of level and 7 d, 21 d of retr	GCF vol greater on 7 d and 21 d of level and retr	NM	IL-8 stat sign dec on 7 d of level
					IL-6-N change				
					IL-8-dec				
32	RANKL, OPG	Mann-Whitney U	NM	NM	RANKL - inc OPG - dec	24 h	*In vitro* compres F for 3, 6, 9, 12, 24, 48 h, RANKL was sign inc in stress (+) grp	N sign diff in mean vol of GCF at 24 h b/w exp T and cont T	Mean RANKL values after 24 h in Exp > cont-mean OPG values after 24 h in Exp < cont
33	IL-1β, SP, PGE2	Paired *t* multiple linear regression analysis	Y	NM	Inc	1 d	VAS inc sign 1 h and 24 h after insert of sepr and returned to bas after 7 d SP and PGE2 sign higher at 1 d and 7 d	NM	IL-1β of exp > cont at 1 h, 1 d, 7 d
34	SP, IL-1β	Mann-Whitney U Spearman’s signed rank	NM	NM	Inc	NM	Avg amt of TM was 1.5 ± 0.4 mm over 168 h-N sign diff in tot prot level at any of exp time periods mean SP levels inc after 8, 24, and 72 h in Exp	N sign diff in mean GCF vol b/w exp and cont T	mean IL-1β levels inc after 8, 24, and 72 h in Exp
35	IL-1β, IL-1RA	ANCOVA	Y	NM	NM	3 d	Inc lag phase with Mx C moved by 4, 13, and 26 kPa. By 52 kPa, distinct lag phase at 3 d, 14 d	Vt vs avg AI in GCF from D sites of Mx C showed a + ve relationship (R^2^ = 0.44)	mean AI for C moved with 52 kPa sign > all other mean AIs
36	IL-8	Mann-Whitney U Wilcoxon	NM	NM	Inc	6 d at tension site, and 1st 24 h at pressure site	GCF vol greater at tension and pressure sites at 24 h and 30 d	NM	IL-8 at both sites inc at 1 h of F. B/w 24
									hand 6 d, inc at tension site. IL-8 inc
									among grps b/w 0 & 1 h stats sign
37	IL-1β, PGE2	Intra-grp: ANOVA Intergrp: 1-way ANOVA	NM	NM	Inc	24 h	PGE2 inc at 24 h > BS in CF and IF. PGE2 inc in CF and IF at 24 h > cont In CF,PGE2 > cont at 168 h, then fall. In IF, PGE2 remain high for a wk.	NM	IL-1β inc at 24 h > BS, max at 24 h after 1st act in IF.
									In IF, IL-1β inc 24 h after 1st reactivn > cont
									In CF and IF, IL-1β inc at 24 h > cont
38	PGE-2, IL-6, GMCSF	Paired *t* (intragrp) independent *t* (J and A)	NM	NM	Inc	24 h	Median GCF vol. in J > A. In J and A PGE2 inc at 24 h > BS	NM	InJ, IL-6, GMCSF inc at 24 h > BS
39	IL-1β, βG	1-tailed paired Student *t*	Y	NM	Inc	IL-1β - 81 d βG - 46 d	βG - significantly dec 14 d after prophy. Exp > cont at 46 d	NM	IL-1β significantly dec 14 d after prophy. Exp > cont at 4 d, 33 to 81 d
40	IL-1β, IL-1RA, AI	Least square regression Pearson product-moment Correl coefficient	NM	NM	Inc	3 d	C retr at 1.27 and 0.87 mm/m for 13 and 4 kPa of stress, resp	+ve corr of velocity and AI from D > M.	IL-1β at M > Cont (13 kPa) IL-1RA at D > M and Cont (4 and 13 kPa) Tot prot at M and D > Cont (4 and 13 kPa)
41	TGF-β	Student’s *t*	Y	NM	Inc	24 h	C retr was 1.1 at 0.1 mm/168 h	NM	TGF-β at exp site at 24 h > bas, Cont
42	IL-1β, IL-6, TNF-α, EGF, β2-μG	Student’s *t*	Y	NM	Inc	24 h	β-2 MG inc at 24 h > BS or 1 h	NM	Intra-grp in Exp: IL-1β inc at 24 h > bas, IL-6 inc at 24 h > bas or 168 h, TNF-α inc at 24 h > bas or 168 h, EGF inc at 24 h > bas
							β-2 MG in exp at 24 h > ant cont		Intergrp btw cont and exp: IL-1β inc in exp > cont at 24 h, mean IL-6 in exp > ant cont, TNF-α in exp at 24 h > ant cont, EGF in exp at 24 h > ant cont
19	IL-1β, PGE	2-way analy of variance paired *t*	NM	NM	Inc	24 h	PGE inc in exp > cont at 24 h, 48 h	NM	IL-1β inc in exp > cont at 1 h, 24 h
							PGE inc at 24 h > BS, 168 h		IL-1β inc at 24 h > bas, 48 h, 168 h
43	IL-1β, TNF-α, NO	Friedman, Wilcoxon test Spearman Rank Correl Analy.	Y	NM	Inc	6 m	PI, GI, PD inc at 1 m > BS, 6 m > BS	NM	IL-1β inc at 6 m > bas
44	RANKL, OPG	Repeated measures ANOVA	NM	NM	Inc	48 h	PM retr −3.73 ± 1.08 mm (laser grp) and 2.71 ± 0.9 mm (Cont grp) Max mean retr v(t) btw 0 and 48 h. Pain intensity pk at 48 h.	NM	RANKL - inc at 48 h > bas
									RANKL/OPG - inc at 48 h > bas
45	RANKL, HSP70,	ANOVA *post hoc* Fisher’s LSD	Y	NM	Inc	24 h	Amylase activity in saliva inc at 24 h at exp > cont	NM	RANKL inc in 24 h > cont
46	MMP-8, IL-1β	Paired-sample *t*	NM	NM	Inc	3 m	NM	MMP-8 dec at 24 h, inc at 3 m	IL-1β inc at 3 m
47	GM-CSF, IF-γ, IL-1β, IL-2, IL-4, IL-5, IL-6, IL-8, IL-10 and TNFα, MMP-9, TIMP-1 and 2, RANKL, OPG	Paired non-parametric Kruskall-Wallis. Spearman Rank Sum analy.	Y	NM	Inc	4 h	+ve corr of GCF vol and plaque index at 0 at(t), (comp)	+ve corr of TNF-α, IL-1β, IL-8, GM-CSF MMP-9 and TIMP levels to speed of OTM at 4 h in Exp corr of IL-1β, IL-8, TNF-α inc to plaque-induced inflam at 0 at (comp).	Exp - IL-1β, IL-8, TNFα inc from 4 h to 42 d. RANKL - inc after 42 d
							Exp - TIMP 1 and 2 inc at 4 h, 7 d and 42 d, MMP-9 inc at 4 h, 7 d.		
							Compr-MMP −9 inc at 4 h and 7 d. TIMP-1 inc at 4 h and TIMP-2 at 7 d.		
							Exp > cont: MMP-9 inc at 0, 4 h at compr, TIMP-2 inc at 0 at Exp, compr		
48	RANK, OPG, OPN, TGF-β1	Friedman Dunn’s multiple comparisons as *post hoc*	Y	NM	Inc	7 d	NM	NM	RANK - inc in 7 d in exp, compres > cont OPG - cont > compres site at 24 h. TGF-β - inc in compres > cont at 7 d.
49	MMP-3, MMP-9, MMP-13, MIP-1β, MCP-1, RANTES	Friedman, Mann-Whitney	Y	NM	NM	NM	MMPs inc at 1 h, dec at 24 h. GCF vol at (comp) > (t) at 21 d	NM	NM
50	IL-1β, SP, PGE2	SPSS 13.0 paired *t*-test Wilcoxon paired signed rank	NM	NM	Inc	1 d	Exp > Cont:	NM	Exp > Cont:
							At D, SP inc at 1 d, 7 d		At M, IL-1β inc at 1 min, 1 h, 1 d, 7 d.
							At D, PGE2 inc at 1 min, 1 h, 1 d, 7 d.		At D, IL-1β at 1 h, 1 d, 7 d.
							At M, PGE2, SP, inc at 1 min, 1 h, 1 d, 7 d > BS.		Exp > bas: At M, IL-1β inc at 1 min, 1 h, 1 d, 7 d. At D, IL-1β inc
							At D, SP, PGE2 inc at 1 d, 7 d > BS.		at 1 h, 1 d, 7 d.
51	IL-1β	Wilcoxon signed-rank Mann-Whitney U	NM	NM	Inc	24 h	IL-1β at 24 h, 48 h at exp > implant	NM	IL-1β inc at 24, 48 h > bas IL-1β dec after 24 h, at 48 h, 168 h, 14 d, 21 d.
52	IL-2, IL-6, IL-8	1-way ANOVA ( interG) Dunnett’s *t* Tukey’s	NM	NM	Inc	24 h	IL-8 inc at 24 h, 48 h in MS grp	NM	IL-2 inc at 24 h > bas
							IL-6 inc at 3 m in MS grp		IL-8 inc in Exp at 1 h, 24 h, 48 h
53	CCL-2 (MCP1), CCL-3, CCL-5 (RANTES), IL-8 (CXCL8), IL-1α, IL-1β, IL-6, TNF-α	ANOVA Tukey’s *post hoc* paired and unpaired *t*	Y	NM	Inc	24 h	MOPs inc the rate of C retr by 2.3-fold compd to cont and contr C - VAS sign at 24 h for Exp and Con	MOPs sign inc cyt and chemokine expression	All cyt and chemo inc in both Cont and Exp at 24 h IL-1 inc also at 28 d
54	TNF-α	Paired *t* (intra G) 1-way ANOVA (interG) Dunnett’s *t* Tukey’s	NM	NM	Inc	24 h	NM	NM	TNF-α inc at 24 h
55	TNF-α, IL-1β, IL-8	1-way ANOVA Paired student t	NM	NM	TNF-α, IL-1β, IL-8 inc in 1 to 2 d of level	1 d	GCF vol inc in 1 to 3 d of leveling	NM	TNF-α, IL-1β, IL-8 inc in 1 to 2 d of level
56	IL-1β, IL-1RA, AI	Least square Regression Pearson product-moment Correl coefficient	NM	NM	AI = 1, then v(t) is not zero	NM	C retr at day 84 for 13, 26, 52 kPa were 4.14 ± 0.19, 6.36 ± 1.32, 5.66 ± 1.38 mm resp	v(t) affected by AI in GCF, stress and IL-1 gene cluster	Faster v(t) seen in 26 kPa, higher GCF and allele 1 homozygosity
57	RANKL, OPG, IL-1, IL-1RA, MMP-9	Repeated measures models	Y	NM	IL-1/IL-1 + IL-1RA - dec RANKL/RANKL + OPG - inc	A - 6 wk	NM	NM	IL-1RA in adults exp > cont at 3 wk. Dec in ratio of (IL-1/[IL-1 + IL-1RA]). Inc in ratio of RANKL to OPG (RANKL/[RANKL + OPG]). RANKL to OPG inc at 6 wk in ado, at 3 wk in A, OPG in ado at Exp < cont at 6 wk
						Ado - 3 wk			
58	IL-2, IL-4, IL-6, IL-8, IL-10, GM-CSF, IFN-γ, TNF-α, MCP-1, IP-10	Stepwise regression	NM	NM	NM	NM	NM	IL-6 levels at bas predictive of GCF flows after 1 y of ortho t/t	No sign change
59	OPG, RANKL	2-way ANOVA. A Bonferroni f	NM	NM	OPG inc in M, dec in D at 1 h	1 h	NM	NM	OPG inc on M, dec in D at 1 h

*Ref No.* reference number, *Stats* statistically, *analy* analysis, *appld* applied, *Confd* confounders, *reg* regulation, *Pk* peak, *Sec* secondary, *outcm* outcome, *correl* correlation, *sign* significant, *Y* yes, *N* no, *NM* not mentioned, *inc* increase, *dec* decrease, *fluct* fluctuated, *h* hour, *m* month, *tot* total, *prot* protein, *conc* concentration, *mg* milligram, *ml* milliliter, *g* gram, > greater than, < less than, *VAS* visual analog scale, *C* canine, *mov* movement, *b/w* between, *CF* continuous force, *IF* interrupted force, *and* and, *F* force, *Assoc* associated, *gen* genetic, *GCF* gingival crevicular fluid, *compd* compared, *bas* baseline, *IL* interleukin, *βG* beta glucoronidase, *TNF-α* tumor necrosis factor alpha, *SD* short duration, *LD* long duration, *Diff* difference, *vol* volume, *retr* retraction, *correl* correlation, *inflam* inflammation, *Avg* average, *cyt* cytokine, *chemo* chemokine, *knwn* known, *MOPs* micro-osteoperforations, *PI* plaque index, *BOP* bleeding on probing, *exp* experimental, *cont* control, *Exp* experimental tooth, *ant* antagonistic, *Avg* average, *Mx* maxilla, *contr* contralateral, *differen* differentiation, *sepr* separator, *grp* group, *compres* compression, *kPa* kilopascal, *max* maximum, *grw* growth, *T* tooth, *Oc* osteoclast, *RDG* rapid canine distalization group, *HG* hybrid reactor group. *v(t)* velocity of tooth movement, *M* mesial, *D* distal, *level* leveling, *retr* retraction.

*CSFs* colony-stimulating factors, *IFNs* interferons, *MCSF* macrophage colony-stimulating factor, *SP* substance P, *IL-1β* interleukin-1 beta, *TNF-α* tumor necrosis factor-alpha, *TGF β* transforming growth factor-beta, *OPG* osteoprotegerin, *OPN* osteopontin, *RANKL* receptor activator of nuclear factor kappa-B ligand, *RANK* receptor activator of nuclear factor kappa-B, *GM-CSF* granulocyte-macrophage colony-stimulating factor, *βG* beta glucuronidase, *PGE2* prostaglandin E2, *IL-1RA* interleukin receptor antagonist, *MCP* monocyte chemoattractant protein, *MMP* matrix metalloproteinases, *MIP* macrophage inflammatory protein, *TIMP* tissue inhibitor of metalloproeintases, *HSP* heat shock proteins, *NO* nitric oxide, *AI* activity index.

The inclusion and exclusion criterion were as under.

Inclusion Criteria: Participants/population -Human studies, age groups (if specified), male to female ratio (if specified), controls (either internal where baseline levels are taken as control or external where contralateral or antagonistic tooth is taken as control), sample size >5 (refers to sample size not number of teeth studied).
  Intervention(s), exposure(s)-Studies on cytokines [including interleukins (ILs), tumor necrosis factor (TNF) and growth factors (GFs), colony-stimulating factors (CSFs), interferons (IFNs)], chemokines, receptors and their antagonists (RANK, RANKL, OPG, OPN) with specified orthodontic mechanics, proper oral hygiene control, no use of antibiotic/anti-inflammatory drugs before or during orthodontic force application, GCF sample collection via periopaper or micropipette placed in sulcus.
Exclusion criteria Participants/population- In vitro studies, animal studies, sample size <5, no control. Intervention(s), exposure(s)- Studies on mediators other than cytokines or chemokines or receptors, cytokine or chemokine or receptor measurement in periodontal tissue and not GCF, cytokine or chemokine or receptor levels consequent to periodontal inflammation and not orthodontic force application, cytokine levels measured in peri-implant fluid.

This is a followed data extraction by two reviewers (PK and NM). The data was recorded in a tabular form based on the following criteria:

1. Participant characteristics (Table 1): number of study subjects (not the number of teeth), teeth considered for study (if specified), sites (if mentioned), age of study subjects (either range or mean age ± standard deviation (SD)), sex, controls, and studied mediators. Apart from these, following inclusion criteria were also considered (if mentioned) no history of drug intake, no bone loss, no gingival inflammation, and pocket depth <2 mm.
2. Study characteristics (Table 2): these were nature of applied force, force magnitudes, force reactivations (if studied), total study duration, observation intervals, and type of tooth movement.
3. Oral hygiene regimen and assessment of gingival health (Table 3): recommendation and frequency of mouthwash intake, oral prophylaxis schedule, use of indices for gingival and periodontal assessment, and their frequency.
4. GCF characteristics (Table 4): time, room temperature and humidity during GCF collection, site, method of collection of GCF, storage and handling characteristics, and technique of mediator analysis.

Quality assessment of the articles included in the review was done based on a Quality Assessment Instrument (QAI) modified and developed from relevant articles in literature [20,21] given in Additional file 2: Annexure 2.

Thirty stringent criteria for evaluation of quality included relevant study design (N = 19), study measurements (N = 3), statistical analysis (N = 5), study results, and conclusions (N = 3). (Additional file 2: Annexure 2). For objective assessment of quality determination, a scoring system was incorporated where scores of 1 to 10 were considered minimal, 10 to 20 were considered moderate, and 20 to 30 were considered highly sensitive. QAI revealed 26 moderately sensitive and 13 highly sensitive studies. None of the studies fell in the score of 1 to 10.

The results were compiled after grouping of observations from similar studies to arrive at conclusions with relevant clinical implications.

## Results

Thirty-nine shortlisted studies [19,22-59] were scrutinized for inclusion and exclusion criteria. Two studies that evaluated mediators both in peri-implant crevicular fluid (PMICF) as well as GCF were included [51,54] but one study that evaluated the levels of mediators directly in sulcus and not GCF was excluded [60].

The studies were categorized based on participant characteristics (Table 1), study characteristics (Table 2), oral hygiene regimen and assessment of gingival health (Table 3), and GCF characteristics (Table 4). All studies displayed control, either internal/external. The levels of biomarkers assessed at baseline level (0 day) were taken as control in the former while in the later, contralateral or antagonistic teeth were taken as control. GCF sampling was done with either using periopaper or micropipette that were placed in the gingival sulcus. An overview of the results obtained has been summarized in Table 5.

### Sample characteristics

#### Sample size

Of the 39 studies, the sample size varied, smallest being 7 subjects [40] to a maximum of 84 subjects [38]. The studies were categorized in four groups, with sample size up to 10 (N = 15), 11 to 20 (N = 16), 21 to 30 (N = 5), and 31 and above (N = 3). Average sample size taken was 10 subjects (N = 10).

### Sex predilection

Information on sex of the subjects was mentioned in N = 36 studies. One study (N = 1) included only male subjects. Equal numbers of male and female subjects constituted the sample in ten studies (N = 10).

### Age distribution

Age was expressed as either range or as mean with standard deviation. There was no mention of age in one (N = 1) study. Comparative evaluation of juvenile and adults was reported in two studies (N = 2) and adolescent vs. adults in one study (N = 1). Age groups of male and females subjects were managed separately in three studies (N = 3). Age group of up to 15 years was considered in 19 (N = 19) studies and 15 years and above was considered in 20 (N = 20) studies. One study (N = 1) considered a large age group interval of 18 to 45 years [53].

### Mediators of orthodontic tooth movement

Cytokines, receptors and their antagonists included in the review have been listed in Table 1. The cytokines have been studied singularly or in combination with other mediators. The most often studied cytokines in tooth movement are IL-1β (N = 21), TNF-α (N = 10), IL-8 (N = 8), IL-6 (N = 8), IL-2 (N=4), IL-4, IL-10 (N = 2), IL-1, IL-5, IL-1α (N = 1), OPN (N = 1), and RANKL (N = 7).

Receptors and their antagonists have been studied in the frequency of OPG (N = 8), IL 1RA (N = 5), and RANK (N = 1). Chemokines have been studied in the order of monocyte chemoattractant protein (MCP)-1 (N = 3), RANTES (N = 2), and IP-10 (N = 1). Studies related to growth factors were GMCSF (N = 3), TGF-β (N = 2), and IFN-γ (N = 2) and there was only one (N = 1) study on Leptin.

### Time period and observation intervals

The total time duration for studies exhibited large variation from as low as 24 h to as high as 70 weeks. Studies were performed for a duration of 24 h (N = 3), 1 week (N = 10), 2 weeks (N = 2), 3 weeks (N = 2), approximately 1 month (N = 5), 2 months (N = 1), approximately 3 months (N = 10), 4 months (N = 2), 5 months (N = 1), and 6 months and above (N = 4).

GCF collection was done at multiple observation times ranging from a maximum of 11 times (N = 2) to minimum at 2 times (N = 4). Nine studies (N = 9) used 4 observation times; eight (N = 8) studies used 6 observation times; four (N = 4) studies used 2, 5, and 7 observation times; three (N = 3) studies used 3, 9, and 10 observation times; and two (N = 2) studies used 8 observation times.

Thirty-seven (N = 37) studies took observation point before activation as ‘zero’ or baseline. The protocol followed for GCF collection was 0, 1 h, 24 h, and 7 days (N = 14) and of 0, 1 h, and 24 h (N = 15). An additional observation point at 14 days (N = 7) and 21 days (N = 6) was also considered. An internal control (baseline levels) was considered in N = 12 studies while other studies (N = 27) took an external control that was either contralateral or antagonistic tooth or tooth other than experimental tooth.

### Study design

#### Mechanics of force application

Twenty-seven studies considered retraction of canine (N = 27) which included NiTi coil spring (N = 18), vertical loop (N = 5), screw-based retractors (N = 3), and segmental mechanics (N = 14). Other methods of force applications were separators (N = 4), expansion with hyrax screw (N = 2), labial tipping with offsets in wire (N = 2), bracket placement (N = 1), and leveling of arches (N = 5).

#### Type of force application

Twenty-six studies (N = 26) used continuous force and 12 (N = 12) employed interrupted force. There was no mention of type of force in two (N = 2) studies. Four studies used continuous and interrupted force on different index teeth (N = 4).

#### Levels of force

Twenty-eight studies (N = 28) mentioned levels of force applied while eleven studies (N = 11) have no mention of it. One-hundred fifty-gram force was used in eight studies (N = 8), followed by 250 g (N = 5) and 100 g (N = 4). One study (N = 1) each employed 50, 120, 130, and 200 g; 70 cN; and 2 to 2.5 N of force. A range of force was applied from 4 to 13 kPa (N = 1), 90 to 115 g (N = 1), and 4, 13, 26, 52, or 78 kPa in N = 2 studies. Force reactivation was considered in three (N = 3) studies to compare continuous and interrupted force.

#### Oral hygiene regimen and gingival health assessment

Professional oral prophylaxis was performed before treatment (N = 28) and at every observation point (N = 23) but was not mentioned in the remaining studies. Oral hygiene regimen with recommendation of chlorhexidine mouthwash was mentioned in 11 studies (N = 11) and its frequency (N = 10) studies. Indices for assessment of gingival and periodontal health were employed before the treatment (N = 30) and at every observation point in N = 24 studies.

#### *GCF characteristics*

The GCF samples were collected at a particular time of the day (N = 11), preferably 9 AM to 12 PM and early morning (N = 2). Twenty-eight (N = 28) studies had no mention of time for GCF collection. The room temperature conditions were considered in six (N = 6) studies and humidity in five (N = 5) studies.

The sites for GCF sample collection were either mesial (N = 5) or distal (N = 21) or both mesial and distal (N = 12). GCF collection was done by periopaper (N = 33) studies, filter paper (N = 3), or filter membrane (N = 2). One study (N = 1) did not mention the technique by which GCF was collected.

Depth of insertion of paper for GCF collection was mentioned in 27 studies with the most common practice being a 1-mm depth (N = 26). One study used 2-mm depth of insertion. Duration of GCF collection was specified to be 30 s in most studies (N = 29) followed by 60 s (N = 4), 3 min (N = 1), 10 s (N = 1), and 20 s (N = 1). Repeated measurements were considered in 19 studies (N = 19) with collection repeated once in N = 11 studies, twice in N = 5 studies, and 3 times in N = 3 studies. The interval was 60 s (N = 14), 90 s (N = 1), and 120 s (N = 1).

The samples were stored at −20°C (N = 7), −30°C (N = 6), −70°C (N = 10), and −80°C (N = 9). The GCF from periopaper was retrieved by Periotron (PT)8000 (N = 18), PT6000 (N = 5), PT (N = 2), or electronic scale (N = 3) and was not mentioned in some studies (N = 11). Mediators were analyzed by ELISA (N = 27), immunoassay (IA) (N = 8), Luminex multianalyte technology (LMAT) (N = 1), Bio-Plex human cytokine assay (BHCA) (N = 1), custom protein array (CPA) (N = 1), or Quantibody Ar kit (QAK) (N = 1). Protein concentration in GCF was measured in pg/μl (N = 12), pg (N = 5), pg/mg (N = 1), pg/ml (N = 5), pg/mg (N = 1), pg/μg (N = 30), pg/30 s (N = 2), pg/20 s (N = 1), and pg/site (N = 1) and was not mentioned in N = 3 studies.

The total number of studies included in this review are 39. However in the result section, some of the variables showing number of studies may be more than 39, as few parameters have been divided into subgroups that have been considered as a separate variable.

#### Mediator levels in GCF

##### Interleukins

Twenty-one studies on IL-1β were evaluated. Of these, ten studies (N = 10) reported that the peak levels of IL-1β were attained at 24 h [19,22,27,33,37,42,50,51,53,54]. The peak levels in other studies have also been reported at 4 h [47], 3 days [35,40], 7 days, 21 days [29], 2 months [22], around 3 months [39,46], and 6 months [43]. One study mentioned peak for different teeth at different observation points [26] that resulted in peak for molars at 67 days, for premolars at 32 days and for central incisors at 39 days. Another study did not mention peak but fluctuation in IL-1β levels on application of different stresses of teeth that were correlated with velocity [24].

One study mentioned IL-1β levels on application of 150-g force to be twice that on application of 50-g force at 24 h and 2 months [22]. Studies have shown decrease in levels of IL-1β, 14 days after prophylaxis, followed by an increase upon activation of orthodontic appliance (N = 2) [26,39]. Forces of short duration show an increase in IL-1β at 7 and 21 days [29]. Levels of IL-1β in experiment teeth were shown to be greater than control teeth at 1 h [19,33], 4 h [47], 8 h [34], 24 h [19,33,34,42],72 h [34], 7 days [33], 25 days [26,39], 32 days [26,39], 33 days [39], 39 days [26,39], 42 days [47], 46 days [26,39], 67 days [26,39], and 81 days [26,39].

A comparison of continuous and interrupted force was evident in one study [37] where it was shown that IL-1β levels in continuous force is greater than baseline at 24 h while in interrupted force, levels were greatest at first reactivation.

Difference in IL-1β levels according to site specification was mentioned in one study [40] where levels at distal site of tooth retraction were greater than mesial site at both 4 and 13 kPa of force application. Placement of elastic separators in molars led to an increase in levels at 1 min, 1 h, 24 h, and 7 days [50] at mesial site while at distal site, increase was seen at 1 h, 24 h, and 7 days [50].

An upregulation in IL-1β levels from baseline levels was evident at 1 min [50], 1 h [50], 24 h [19,37,42,51,54], 48 h [51], 7 days [50], 3 months [46], and 6 months [43] and downregulation was seen in 48 h, 168 h, 14 days, and 21 days [51].

For IL-6, the levels were found to increase at 24 h [38,53] when continuous forces were applied for retraction or tipping.

IL-8 also increased on application of continuous force for retraction at 1 h [36,52] both on tension and pressure sites [36] and also at 4 h [47], 24 h, and 48 h [52]. Placement of separators led to an increase in levels at 24 h [27]. Fall in levels was observed at 7 days of leveling and an increase was seen at 7 days, 21 days of retraction [31].

##### TNF-α

Application of interrupted force witnessed an increase in levels at 1 h [28] and 24 h [27,42] while continuous force application led to increase in levels at 24 h [53,54] or 4 h to 42 days [47]. There was a decrease in levels in 1 week on continuous force retraction by hybrid retractor (HG) [28]. TNF-α levels also increased at 1 day [54], at 3 months [29] of leveling, and at 6 months, just before retraction [29]. A comparison of continuous force application by hybrid retractor (HG) by HG to interrupted force by rapid canine distalizer (RDG) showed higher values at 1 h in RDG group compared to HG [28].

### Levels of receptor and their antagonists in GCF

#### *RANKL*

RANKL showed an increase in levels at 24 h [30,32,45] greater than control as well as baseline with specific mention of levels in juveniles and adults [30,32]. Two studies (N = 2) mentioned increase in levels greater than baseline, one at 48 h [44] and other at 42 days [47] at 24 h greater than control [45], at 48 h [44], 42 days [47] greater than baseline. Correlation with age was established with levels in adults being less than juveniles [30,32], an increase in RANKL/OPG ratio in 6 weeks in adolescents [57].

#### IL-1RA

Lower value IL-1RA was shown to be associated with higher velocity of tooth movement (Vt) [24]. It was also a determinant of activity index (AI) that is ratio of concentrations of IL-1β and IL-1RA in GCF, known to correlate with Vt [24,35,40,56]. One study mentioned distinction of site where levels at distal site of retraction were greater than mesial and control on application of 4 and 13 kPa force [40]. A comparison between levels in adults and adolescents revealed a decrease in ratio of IL1/(IL1 + IL-1RA) in 3 weeks [57].

#### *OPG*

The levels were decreased in experimental teeth at 1 h [25], 24 h [25,30,48], 168 h [25], 1 month [25], and 3 months [25] than baseline levels. A distinction of age-specified levels of OPG as well as ratio of RANKL/OPG was found to be lower in adults than in juveniles [30]. The levels of OPG in experimental teeth were found to be lower in adolescents in 6 weeks compared to control teeth [57]. Its values were less in adolescents in 6 weeks [57]. Site specification determined levels to increase on tension site (mesial) and to decrease on compression site (distal) [59].

*Chemokines* [CCL-2 (MCP1), CCL-3, CCL-5 (RANTES), IL-8 (CXCL8)] showed an increase in both experimental and control teeth at 24 h of force application [53]. Levels of IL-8 were increased when force was applied for a short duration in separator placement [27], or longer duration in initial alignment [27], 1 to 2 days of leveling [54], increase from 4 h to 42 days [47], at 1 h, 24 h, and 48 h [52] when continuous forces were applied for retraction. Levels also showed a decrease in 7 days of leveling [31]. A difference in levels was observed with distinction of site, with levels increased at both mesial and distal sites at 1 h of force application, between 24 h and 6 days at tension site and a statistically significant increase at tension site greater than compression site [36]. Two studies (N = 2) on chemokines did not reveal any significant findings [49,58].

## Discussion

This systematic review was primarily aimed to conjure substantial evidence regarding the cytokine, chemokine, receptor and their antagonist (RANK, RANKL, OPG) levels in GCF consequent to application of orthodontic force. The literature revealed heterogeneity in study designs pertaining to participant characteristics, force application, levels of force, GCF collection methods and collection protocol, storage, and oral hygiene maintenance regimen. To draw logical conclusions each of the variables was tabulated and analyzed separately. Associations of change in levels of mediators were established with mechanics of applied orthodontic force, amount of force, force reactivations, differentiation in levels between tension and compression sites, age groups (juveniles and adults, growers and non-growers), and velocity of tooth movement (Vt).

The altering levels, rise, and fall of the mediators in GCF are suggestive of underlying intricate biological remodeling processes in bone and periodontal tissues that eventually leads to OTM. The forces employed for OTM or midpalatal expansion led to an initial increase in levels of bone-resorptive mediators as well as associated receptors namely IL-1β, IL-8, RANKL, and TNF-α as early as 1 min [50] or 1 h [28] and attained peak in 24 h [19,22,27,30-33,37,42,45,50,51,53,54]. These mediators slowly decrease to baseline in subsequent observation points at 48 h, 168 h, 14 days, and 21 days [28,31,51]. On the contrary, bone-forming mediators like OPG show an immediate decrease in levels on application of orthodontic forces at 1 h on distal site of retraction [25,59], at 24 h [25,30,32,48]. The role of RANK, RANKL, and OPG system in governing osteoclastogenesis has also been corroborated in animal studies [61,62] and *in vitro* studies [63-66] on periodontal ligament cells. When compressive orthodontic force is applied, upregulation of RANKL occurs which leads to stimulation of PGE2 pathway and finally, osteoclastic activity is initiated which results in bone resorption [65,66]. OPG, a RANKL decoy receptor generated by osteoblastic cells and cells of the periodontal ligament, binds to RANKL and inhibits RANK/RANKL interaction that is the mainstay of osteoclastogenesis [67].

Besides these receptors, other factors that are directly or indirectly responsible for differentiation, survival, and activity of osteoclasts are cytokines (IL-1β, TNFα, IL-6) and chemokines (CCL2, CCL3, CCL5, CCL7, CCL9, IL-8) [68,69]. The literature search in the present review found an increase in levels of these mediators in GCF on orthodontic force application. Evidence proves that mechanical stress induces acute inflammatory changes that alter the microvascular environment, with studies supporting local release of mediators IL-1β, TNF-α, and expression of chemokines that ultimately promotes leukocyte adhesion and migration [70]. IL-1β (N = 21) and TNF-α (N = 10) are the most researched cytokines, supporting their role in the inflammatory changes associated with orthodontic tooth movement (OTM). Variation in mediator levels with type of force and force reactivations has also been evaluated to study their clinical implications with IL-1β, PGE2, or TNF-α levels showing an initial increase, both in continuous and interrupted force [28,37]. However, timely reactivations in interrupted force led to an upregulation of these mediators, indicative of greater inflammation than on continuous force application [28,37]. This finding is in accordance with other studies that support association of light continuous forces for OTM with minimal inflammation, root resorption, and hyalinization of the periodontal ligament [71-73]. More recent techniques for accelerated orthodontics like micro-osteoperforations have also conducted studies at cellular level that led to increase in GCF levels of cytokines ( IL-1α, IL-1β, IL-6, TNF-α) and chemokines at 24 h, giving evidence of underlying inflammatory process associated with inducing perforations in cortical bone [53]. An animal study further supports the release of proinflammatory cytokines with micro-osteoperforations, known to recruit osteoclast precursors and hence increase OTM by influencing the bone remodeling process [74].

Results of the review showed that compression side witnessed a decrease in bone-formative OPG by 24 h [48] and increase in bone-resorptive RANK and TGF-β1 after 7 days [32,48]. Other mediators showing temporal variation on compression side were IL-1β that increased as early as 1 min [50] or after 4 h [47], RANKL after 42 days [47] or after 24 h both in juveniles and adults [30], and IL-8 after 4 h [47] or after 10 days [36]. In contrast, the tension site showed an appreciable increase in TNF-α [47] and other bone-resorbing mediators like IL-1β, PGE2, and IL-8. But the rise occurred earlier than compression and at all observation points with levels higher than at the compression site [36,47]. This difference is hard to understand as the concept of compression on one side and tension on the other side of the tooth undergoing movement is hypothetical. It has been logically contradicted since the anatomical shape and surface morphology of the tooth root cannot be considered confined to definite geometry. Thus, forces when applied lead to biological response in whole of the periodontal apparatus that cannot be differentiated for release of inflammatory mediators in GCF which is a freely circulating fluid in gingival sulcus. Therefore, mediator levels in GCF collected from mesial or distal sites of the tooth may not be indicative solely of compression or tension zone activity.

Synopsis of the studies included in the review also revealed that age and growth status were factors influencing the level of cytokines in GCF that is shown to have an effect on the rate and amount of tooth movement. Mediator levels were seen to vary with growth status of individuals as evaluated in adolescents and adults [57] or compared in juveniles and adults [30,38]. In one study, different mediators were found to increase in different age groups with IL-6, GM-CSF increasing only in juveniles while PGE2 increasing both in juveniles and adults [38]. In addition, activity index (AI) that is the ratio of IL-1β/IL-1RA in GCF, was found to influence velocity (Vt) of OTM both in growers and non-growers [35]. Mean Vt of growers was 0.050 mm/day and of non-growers was 0.024 mm/day for the same amount of applied stress that was correlated with higher levels of IL-1β and an increased AI in growers [35]. A greater Vt and amount of OTM in juveniles as compared to adults could be explained on the basis of a higher RANKL/OPG ratio in GCF in juveniles [30]. Other studies also support the variation in Vt according to varying mediator levels and AI in GCF [24,35,40,56]. It was found that association between AI and Vt was stronger from distal than from mesial of retracted teeth, thus emphasizing greater values of IL-1β on the distal [40]. Thus, evidence from this literature review emphasizes the role of RANKL/OPG ratio in OTM in either of the age groups owing to its significance in osteoclastogenesis and bone resorption that ultimately alters the amount and velocity of OTM.

Secondary outcome of this review was the association of intensity of pain with different force levels. The level of IL-1β was seen to increase at 1 day [50] that correlated with increased pain intensity and subsequently, there was a decrease seen in 7 days. Another study suggested pain was less with 50-g force as compared to 150-g force that was correlated with greater levels of IL-1β with application of 150-g force [22]. It can be concluded that 150-g force is marked by higher levels of IL-1β in GCF compared to 50-g force and high pain intensity.

## Conclusions

This systematic review is focused on association of cytokine and receptor levels or activity index in GCF with velocity of tooth movement, nature of force applied, pain intensity, and growth status/age of the subjects, leading to following conclusions:

1. Application of orthodontic forces causes an immediate release of inflammatory bone-resorptive mediators (IL-1β, TNF-α) in 1 h that reach peak in 24 h, thus supporting the role of inflammation in initial OTM.
2. Bone-forming mediators like OPG witnessed a fall in levels immediately after orthodontic force application indicating bone resorption to be the key process in initiating tooth movement.
3. The levels of cytokines decrease after attaining peak values, mostly at 24 h in continuous forces but repeated activations in interrupted force upregulate their secretion.
4. A rise in GCF levels of IL-1β with higher force levels (150 vs 50 g) has been linked to increased pain intensity during OTM.
5. Juveniles exhibiting greater RANKL/OPG ratio and activity index (AI) (IL-1β/IL-1RA) in GCF displayed faster rate of OTM than adults or non-growers.
6. Increased velocity of tooth movement (Vt) has been correlated with a greater activity index (AI) in GCF.

The literature search and critical review have also provided a lead to lacunae of the research in this field. There is lack of uniformity of study design with respect to sample size, age, sex ratio, observation intervals, duration of observations, mechanism employed to initiate OTM and ethnic/nutritional barriers. These are potential confounders which can influence the outcome [75]. A major drawback identified in the current review was the lack of consideration of sex on mediator levels that are known to be sensitive to estrous cycle. Animal studies have evaluated correlation between ovarian activity and PGE2, IL-1β levels in GCF of female cats during OTM. Results revealed that mediator levels of estrous groups were lower than anestrous and ovariectomized groups on 6 and 12 days, indicating that ovarian activity can affect OTM [76]. The threshold levels of the inflammatory mediators for initiation for OTM also remain unexplored. Besides GCF, peri-implant fluid may also be a potential medium to study these markers noninvasively in future studies [77]. Search of pain killers having least effect on bone-resorbing mediator levels as a drug of choice may be a potential area of future research [78]. Research related to the role of mediators in external apical root resorption (EARR) and relapse has also received little attention and are important research areas requiring further exploration.

## Additional files

Additional file 1: Annexure 1. Search Strategy. Additional file 2: Annexure 2. Quality assessment instrument.
