# Supplementary material for: Effect of orthodontic forces on cytokine and receptor levels in gingival crevicular fluid: a systematic review
Source: Prog Orthod. 2014 Dec 9;15(1):65. doi: 10.1186/s40510-014-0065-6 (PMC4259981; doi:10.1186/s40510-014-0065-6)
Supplement: Additional file 1: Annexure 1. — Search Strategy. [file 40510_2014_65_MOESM1_ESM.doc]

**Additional File 1:Search Strategy**

((biomarker)OR (cytokines) OR (interleukin) OR (il-1 beta) OR (IL-2) OR (IL-6) OR (IL-8) OR (interleukin-1 beta) OR (INTERLEUKIN-2) OR (INTERLEUKIN-6) OR (interleukin-8) or( tumour necrosis factor alpha) OR (leptin) OR (interferon)OR(chemokines) OR (Monocyte chemoattractant protein) OR (MCP) OR (RANTES) OR (Macrophage inflammatory protein) OR (MIP) OR (polypeptide growth factors) OR (lymphokines) OR (transforming growth factor) OR (stress proteins)OR (RANKL) OR(OPG)OR (RANK) OR (receptor activator of nuclear factor ᴋ B) OR (receptor activator of nuclear factor ᴋ B ligand) OR (osteoprotegerin)) AND ((orthodontic force) OR (tooth movement)) AND ((GCF) OR (gingival crevicular fluid))
