# Supplementary material for: Effect of orthodontic forces on cytokine and receptor levels in gingival crevicular fluid: a systematic review
Source: Prog Orthod. 2014 Dec 9;15(1):65. doi: 10.1186/s40510-014-0065-6 (PMC4259981; doi:10.1186/s40510-014-0065-6)
Supplement: Additional file 2: Annexure 2. — Quality assessment instrument. [file 40510_2014_65_MOESM2_ESM.docx]

**Additional file 2: Quality assessment instrument**

1. **Study Design (19)**
2. Objective—objective clearly formulated (Y),(No),(Unclear)
3. Sample size—considered adequate (Y),(No),(Unclear)
4. Spectrum of patients representative of patients receiving the test in practice (Y),(No),(Unclear)
5. Ethical clearance mentioned (Y),(No),(Unclear)
6. Selection criteria- clearly described (Y),(No),(Unclear)
7. Randomization–stated (Y),(No),(Unclear)
8. Baseline characteristics- clearly defined (Y),(No),(Unclear)
9. Control- clearly defined (Y),(No),(Unclear)
10. Orthodontic mechanics explained in sufficient detail to permit replication of experiment (Y),(No),(Unclear)
11. Orthodontic force- clearly specified (Y),(No),(Unclear)
12. Description of execution of index test*sufficient to permit replication of test (Y),(No),(Unclear)
13. Absence of time difference between index test & control- mentioned (Y),(No),(Unclear)
14. Index test executed at specified time and environmental conditions (Y),(No),(Unclear)
15. Use of proper indices for assessment of gingival & periodontal status
    1. Pre-treatment assessment (Y),(No),(Unclear)
    2. At each observation time (Y),(No),(Unclear)
16. Oral hygiene regime-mentioned (Y),(No),(Unclear)
17. Prophylaxis done
    1. Pre-treatment (Y),(No),(Unclear)
    2. At each observation time (Y),(No),(Unclear)

**II.Study Measurements (3)**

1. GCF handling characteristics- explained(Y),(No),(Unclear)
2. Measurement method–appropriate to the objective (Y),(No),(Unclear)
3. Reliability–adequate level of agreement (Y),(No),(Unclear)

III. **Statistical analysis (5 )**

1. Dropouts–dropouts included in data analysis (Y),(No),(Unclear)
2. Statistical analysis–appropriate for data (Y),(No),(Unclear)
3. Confounders–confounders included in analysis (Y),(No),(Unclear)
4. Statistical significance level–P value stated (Y),(No),(Unclear)
5. Confidence intervals provided (Y),(No),(Unclear)

**IV.Study results and conclusions (3)**

1. Index test compared to baseline (Y),(No),(Unclear)
2. Index test compared to control (Y),(No),(Unclear)
3. Conclusions- specific (Y),(No),(Unclear)

****Index test: Refers to collection of GCF at each observation interval in treatment teeth***
